# Electrocardiography-based artificial intelligence predicts the upcoming future of heart failure with mildly reduced ejection fraction

**DOI:** 10.3389/fcvm.2025.1418914

**Published:** 2025-02-10

**Authors:** Dae-Young Kim, Sang-Won Lee, Dong-Ho Lee, Sang-Chul Lee, Ji-Hun Jang, Sung-Hee Shin, Dae-Hyeok Kim, Wonik Choi, Yong-Soo Baek

**Affiliations:** ^1^ Division of Cardiology, Department of Internal Medicine, Inha University College of Medicine, Incheon, Republic of Korea; ^2^ Department of Electrical and Computer Engineering, Inha University, Incheon, Republic of Korea; ^3^ DeepCardio Inc., Incheon, Republic of Korea; ^4^ Department of Computer Engineering, Inha University, Incheon, Republic of Korea; ^5^ Department of Information and Communication Engineering, Inha University, Incheon, Republic of Korea; ^6^ School of Computer Science, University of Birmingham, Birmingham, United Kingdom

**Keywords:** artificial intelligence, electrocardiography, heart failure, predictability, ejection fraction

## Abstract

**Background:**

Heart failure with mildly reduced ejection fraction (HFmrEF) has emerged as the predominant subtype of heart failure (HF). This study aimed to develop artificial intelligence (AI)-electrocardiography (ECG) to identify and predict the prognosis of patients with HFmrEF.

**Methods:**

We collected 104,336 12-lead ECG datasets from April 2009 to December 2021 in a tertiary centre. The AI-ECG encompasses a novel model that combines an automatic labelling preprocessing method with a transformer architecture incorporating a triplet loss for HFmrEF analysis.

**Results:**

The receiver operating characteristic analyses revealed that the area under the curve of AI-ECG for identifying all types of HF was acceptable [0.873, 95% confidence interval (CI): 0.864–0.893], while that for identifying patients with HFmrEF was relatively lower (0.824, 95% CI: 0.794–0.863) than that for those with HF with reduced ejection fraction (EF) (0.875, 95% CI: 0.844–0.912) and those with normal EF (0.870, 95% CI: 0.842–0.894). The analysis of ECG features showed significant increases in QRS duration (*p* = 0.001), QT interval (*p* = 0.045), and corrected QT interval (*p* = 0.041) with increasing “Severity by Euclidean distance”. Following the predictability analysis with another group of 953 patients for improvements of follow-up EF in HFmrEF, the patients were grouped into three clusters based on the AI-Euclidean distance; Cluster 1 had the most severe cases and poorer outcomes than Clusters 2 (*p* < 0.001) and 3 (*p* < 0.001).

**Conclusions:**

AI-ECG presents an innovative approach for the prognostic stratification of cardiac contractility in patients with HFmrEF. In patients with HFmrEF, disease progression can be predicted using AI-ECG.

## Graphical Abstract

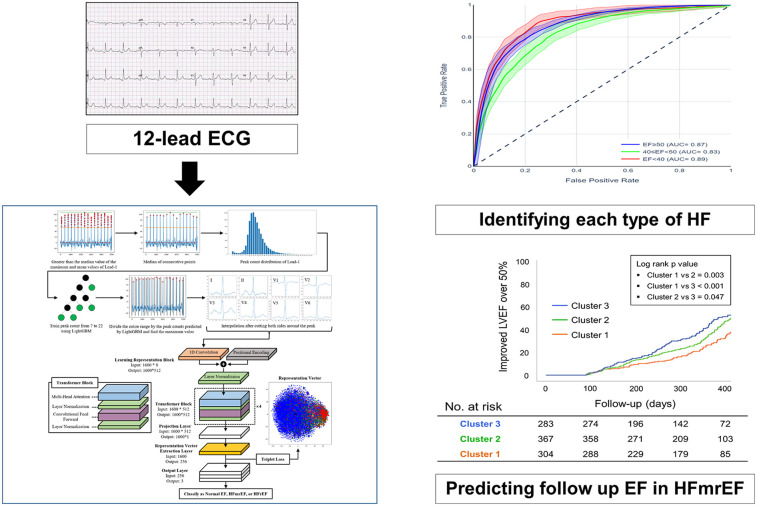

## Introduction

Heart failure (HF) with mildly reduced ejection fraction (HFmrEF) is defined as left ventricular (LV) ejection fraction (EF) between 41% and 49% (1); its prevalence is 10%–25% among patients with HF, and it is currently receiving attention for its clinical implications, including its complexity, heterogeneity, and dynamic features (2). Patients with HFmrEF have unique heterogeneous characteristics that may be enhanced with HF with improved EF (HFimpEF) or may be exacerbated by HF with reduced ejection fraction (HFrEF) (3, 4). Therefore, factors predicting LVEF improvement or deterioration in HFmrEF can be considered important variables (5).

Patients with HFmrEF had a relatively better LV systolic function than those with HFrEF. However, based on previous studies, it is difficult to conclude whether the prognosis of HFmrEF is absolutely better than that of HFrEF (6). Their EFs often change over time compared to those with other types of HF, and patients who progress from HFmrEF to HFrEF show a poorer prognosis than those who remain with the same type of HFmrEF or transition to HFimpEF (7). Therefore, predicting LV contractility before disease progression has significant implications for the clinical prognosis in these populations. The echocardiographic evaluation of LV EF is important in the serial follow-up of patients with HFmrEF. Because the clinical status is often not correlated with the current cardiac contractility, the diagnosis is sometimes delayed, and the regular examination with echocardiography is less effective in terms of time and cost in some patients. Thus, it would be helpful to identify the LV EF before performing echocardiography; however, to date, clinical or diagnostic devices to identify patients with HFmrEF other than echocardiography have not been developed.

Deep learning models have been introduced to detect HF using various electrocardiography (ECG) features (8–10). Several ECG signals have different characteristics according to HF types because their findings are associated with the process of cardiac remodelling and structural changes. It is possible to identify the types of HF using artificial intelligence (AI)-based ECG. AI-ECG has been validated for detecting HFrEF and HFpEF. However, there is a lack of data on identifying HFmrEF and predicting its prognosis using AI-ECG. Therefore, in this study, we aimed to develop AI-ECG to identify HFmrEF and to predict the prognosis of patients with HFmrEF.

## Materials and methods

### Study design and population

**Figure 1:**
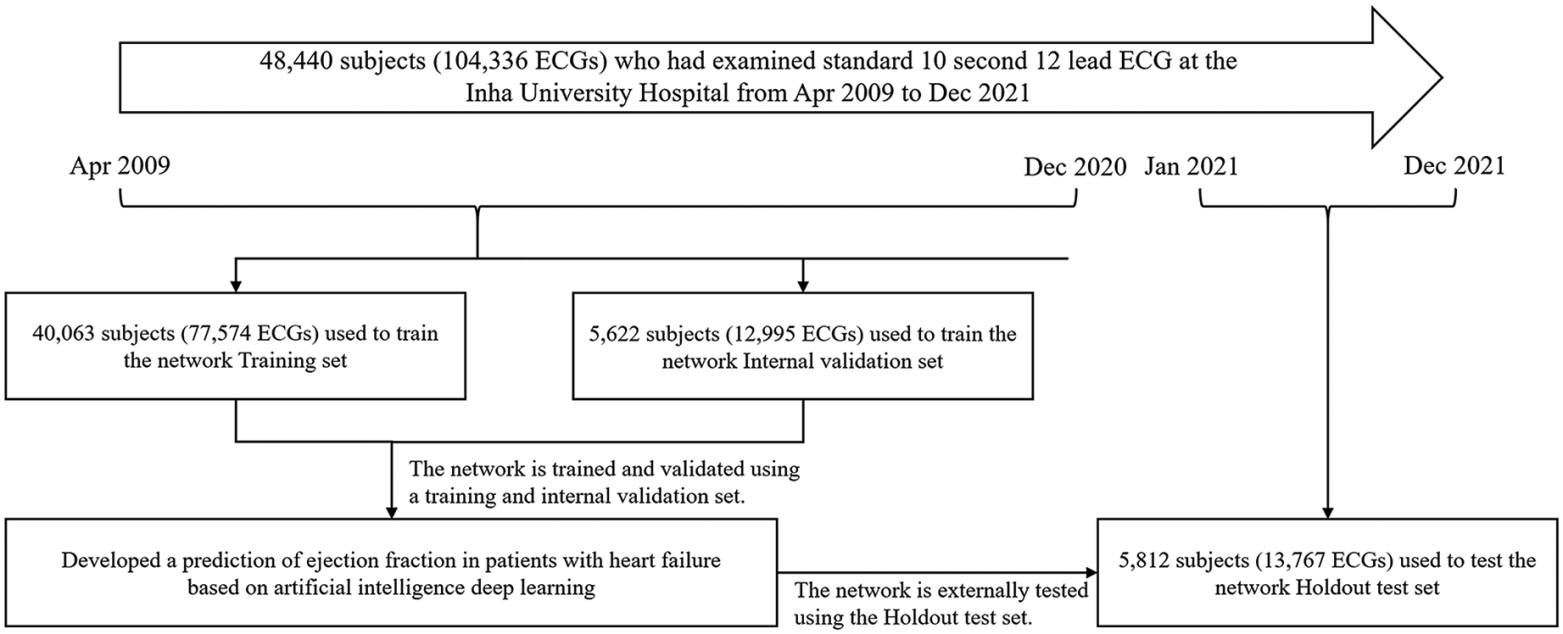
Development, validation, and schematic strategy of the dataset creation and analysis.

The study design is shown in Figure 1. The study population included adult patients aged over 18 years who had performed ECGs within 3 months among those who underwent echocardiography on initial examination for clinical evaluation or health check-up. Among them, we excluded subjects with atrial fibrillation and other non-sinus rhythms such as junctional or cardiac pacing rhythm to simplify the analysis and concentrated on specific signal characteristics within a controlled group. From the 48,440 patients in the study cohort, 104,336 ECGs obtained at a single tertiary centre, Inha University Hospital in South Korea from April 2009 to December 2021 were retrospectively collected and used to develop AI-ECG. According to the current criteria, patients who experienced clinical symptoms of dyspnea that were accompanied by signs of pulmonary edema or effusion on chest x-ray and elevated N-terminal pro-brain natriuretic peptide were defined as HF (11). Among them, patients whose LVEF was 40% or less on initial echocardiography were classified by HFrEF, and those with LVEF between 41% and 49% were classified by HFmrEF. There were 6,819 ECGs for HFrEF, 9,077 for HFmrEF, and 88,440 for a population whose LVEF was ≥50%, regardless of HF symptoms. For all subjects, ECG data between April 2009 and December 2020 were used for training and validation, and those between January 2021 and December 2021 were used for holdout tests. This study was performed in accordance with the Declaration of Helsinki, and the study protocol was approved by the Institutional Review Board of Inha University Hospital (IRB number: 2023-03-024). The institutional review board waived the need for the informed consent of the patients because of the retrospective nature.

### AI-ECG model for mildly reduced EF analysis

Our proposed method is schematically shown in Supplementary Figure 1 and has three novel features. First, we employed a technique for learning the R-peak positions through automatic labelling and machine learning. Second, our deep learning model based on transformer architecture (12) used in BERT (13) and GPT (14) incorporated both triplet (15) and cross-entropy losses to extract a representation vector of the input ECG and enabled the classification of patients with HFrEF (EF ≤40), HFmrEF (41 ≤EF ≤49), and normal EF (EF ≥50%). Third, we analysed the characteristics of HFmrEF ECG from the extracted vectors. The smaller the Euclidean distance between the representation vector of the ECGs of patients with HFmrEF and the average representation vector of the ECGs of those with normal EF, the higher the similarity between the ECGs of the respective patients with HFmrEF and ECGs of most patients with normal EF. Euclidean distance is a measure of the straight-line distance between two points in space, calculated by summing the squares of the differences in each coordinate and taking the square root of the result. Hence, we defined the difference between the Euclidean distance of the representation vector of the ECGs of patients with HFmrEF and that of the average representation vector of those with normal EF and HFrEF as “Severity by Euclidean distance”. Further details on the first and second distinctive features are provided in the Supplementary Material.

Furthermore, we performed an additional analysis to assess the potential of our model to predict whether LVEF improved at over 50% on follow-up echocardiography in patients with HFmrEF. For this analysis, we categorised patients into groups based on the severity of AI-ECG findings, referred to as “Clusters”. We included 953 patients with HFmrEF whose ECG and echocardiographic data were not used for the training, validation, or holdout tests. These patients underwent initial ECG and echocardiography between January 2010 and March 2022. On initial echocardiography, LVEF was 41%–49%, which was compatible with HFmrEF. Regardless of the etiology of HF, patients with HFmrEF had visited the hospital regularly and had been prescribed the optimal medication for HFmrEF. They performed follow-up echocardiography 3–18 months after the initial evaluation. Cluster groupings ranged from 1 to 3, indicating varying degrees of AI-ECG severity, with Cluster 1 representing the most severe.

### Statistical analyses

Continuous variables are presented as the mean ± standard deviation, and categorical variables are presented as percentages and frequencies. The clinical and ECG components were compared using a one-way analysis of variance and *χ*^2^ test for continuous and categorical variables, respectively. The performance of the AI model was evaluated using receiver operating characteristic (ROC) curves to predict the accuracy, sensitivity, specificity, and F1 score of the dataset. The F1 score (balanced F-score) is the harmonic mean of precision and recall. For all variables, statistical significance was set at *p* < 0.05. All statistical analyses were performed using SPSS software (version 25.0; IBM Corp, Armonk, NY, USA).

## Results

### Baseline clinical and ECG characteristics

The baseline clinical characteristics and ECG components of the patients in the training, validation, and test sets are presented in Table 1. The mean age of patients was 63.0 ± 15.9 years, and 55.8% were males. The proportions of patients with hypertension, diabetes mellitus, and stroke were 5.7%, 5.3%, and 5.4%, respectively. The mean LVEF was 59.7% ± 9.5%. On ECG, the ventricular rate per minute was 78.4 ± 19.9, and the PR interval, QRS duration, and corrected QT interval (QTc) were 164.1 ± 28.3, 93.8 ± 17.4, and 442.6 ± 38.5 ms, respectively.

**Table 1:** Characteristics of the patients and laboratory results in the training, validation, and test sets.

Characteristics	All (*n* = 104,336)	Training set (*n* = 77,574)	Validation set (*n* = 12,995)	Test set (*n* = 13,767)	*p*-value
Clinical variables					
Age	63.0 ± 15.9	62.0 ± 15.8	65.5 ± 15.6	66.2 ± 15.7	<0.001
Sex, male, *n* (%)	58,246 (55.8)	42,976 (55.4)	7,380 (56.8)	7,890 (57.3)	<0.001
BMI, kg/m^2^	24.4 ± 11.0	24.8 ± 15.6	24.1 ± 4.4	24.1 ± 4.2	<0.001
HTN, *n* (%)	5,928 (5.7)	3,501 (4.5)	1,388 (10.7)	1,039 (7.6)	<0.001
DM, *n* (%)	5,542 (5.3)	2,891 (3.7)	1,401 (10.8)	1,250 (9.1)	<0.001
CKD, *n* (%)	2,427 (2.3)	1,249 (1.6)	646 (5.0)	532 (3.9)	<0.001
Dyslipidaemia, *n* (%)	2,203 (2.1)	1,222 (1.6)	508 (3.9)	473 (3.4)	<0.001
Stroke, *n* (%)	5,659 (5.4)	2,693 (3.5)	1,505 (11.6)	1,461 (10.6)	<0.001
MI, *n* (%)	3,757 (3.6)	1,792 (2.3)	922 (7.1)	1,043 (7.9)	<0.001
PAD, *n* (%)	898 (0.9)	534 (0.7)	195 (1.5)	169 (1.2)	<0.001
Echocardiography					
LVEF (%)	59.7 ± 9.5	59.7 ± 9.5	59.9 ± 9.4	59.8 ± 9.3	0.120
Electrocardiography					
Ventricular rate	78.4 ± 19.9	78.0 ± 19.5	79.7 ± 20.9	79.7 ± 21.1	<0.001
PR interval	164.1 ± 28.3	163.8 ± 28.1	165.4 ± 28.6	165.0 ± 28.9	<0.001
QRS duration	93.8 ± 17.4	93.9 ± 17.1	93.5 ± 18.2	93.7 ± 18.5	0.037
QT interval	395.0 ± 47.4	394.9 ± 46.5	395.0 ± 49.7	395.9 ± 50.2	0.073
Corrected QT interval	442.6 ± 38.5	441.4 ± 37.7	445.7 ± 40.19	446.39 ± 40.62	<0.001
P axis	48.75 ± 24.51	48.72 ± 24.24	48.56 ± 25.31	49.09 ± 25.31	0.233
R axis	33.02 ± 41.94	33.97 ± 41.36	29.28 ± 41.89	31.16 ± 44.85	<0.001
T axis	50.43 ± 52.41	49.93 ± 50.92	51.36 ± 56.51	52.35 ± 56.45	<0.001

BMI, body mass index; CKD, chronic kidney disease; DM, diabetes mellitus; HTN, hypertension; LVEF, left ventricular ejection fraction; MI, myocardial infarction; PAD, peripheral artery disease.

### Performance of AI-ECG for identifying the phenotypes of HF

**Figure 2:**
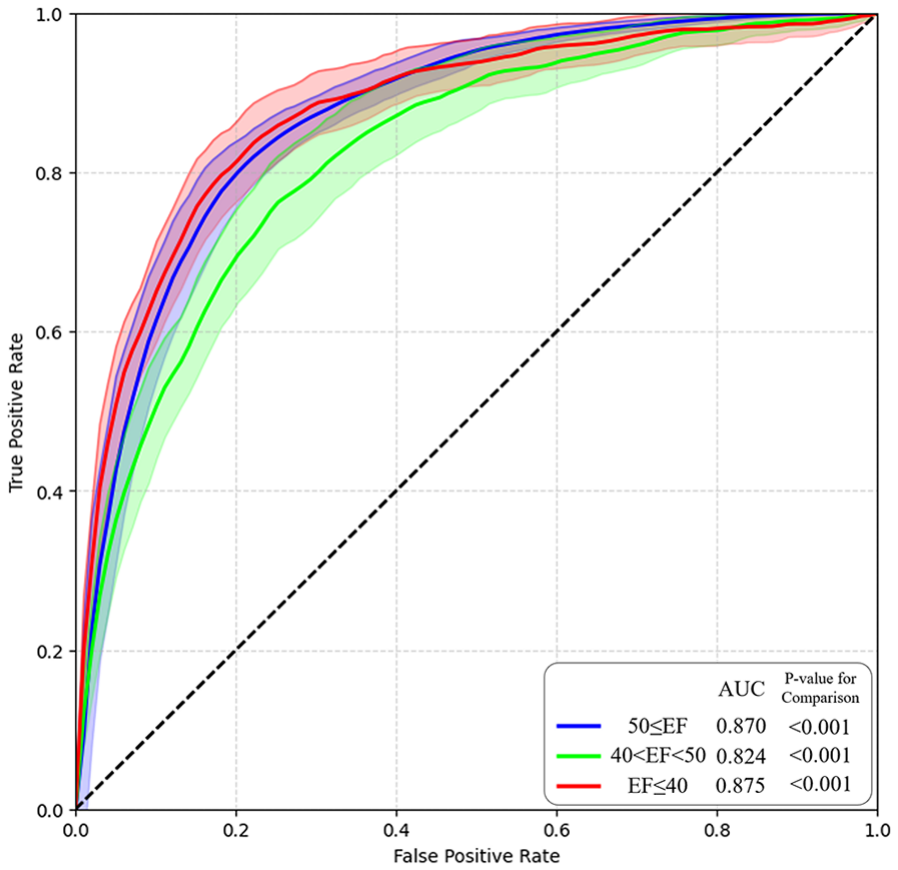
AI model performance and ROC curve. AI, artificial intelligence; ROC, receiver operating characteristic.

We used the validated AI-ECG model to identify LVEF using ROC analyses. First, the predictability of the AI-ECG model was confirmed according to the three phenotypes of HF (Table 2; Figure 2). Predicting each HF phenotype of AI-ECG was acceptable. The overall accuracy was 87.3% (95% CI: 86.4–89.3) in identifying HF type: AI-ECG had the best performance in predicting patients with HFrEF [AUC 0.875, 95% confidence interval (CI): 0.844–0.912], followed by those with normal EF (AUC 0.870, 95% CI: 0.842–0.894) and HFmrEF patients (AUC 0.824, 95% CI: 0.794–0.863). Next, because we confirmed that the model had difficulty accurately classifying HFmrEF in the three-category classification, we also conducted binary classification of patients with LVEF of less than 40% (EF ≤40%) and those with more than 40% (EF >40%). The results showed that the model's areas under the curve (AUCs) for predicting these two groups were 0.86 (95% CI: 0.82–0.92) (Supplementary Figure 2).

**Table 2:** Performance of AI-ECG.

	AUC	TPR = Recall = Sensitivity	TNR = Specificity	PPV = Precision	NPV	F1-score
Macro	0.856	0.531	0.799	0.604	0.854	0.549
Weighted	0.867	0.875	0.522	0.855	0.698	0.860
50 ≤ EF	0.870	0.918	0.451	0.918	0.659	0.940
40 < EF < 50	0.824	0.178	0.983	0.442	0.939	0.254
EF ≤ 40	0.875	0.452	0.963	0.451	0.963	0.452

AI-ECG, artificial intelligence-based electrocardiography; AUC, area under the curve; EF, ejection fraction; NPV, negative predictive value; PPV, positive predictive value; TNR, true negative rate; TPR, true positive rate.

**Figure 3:**
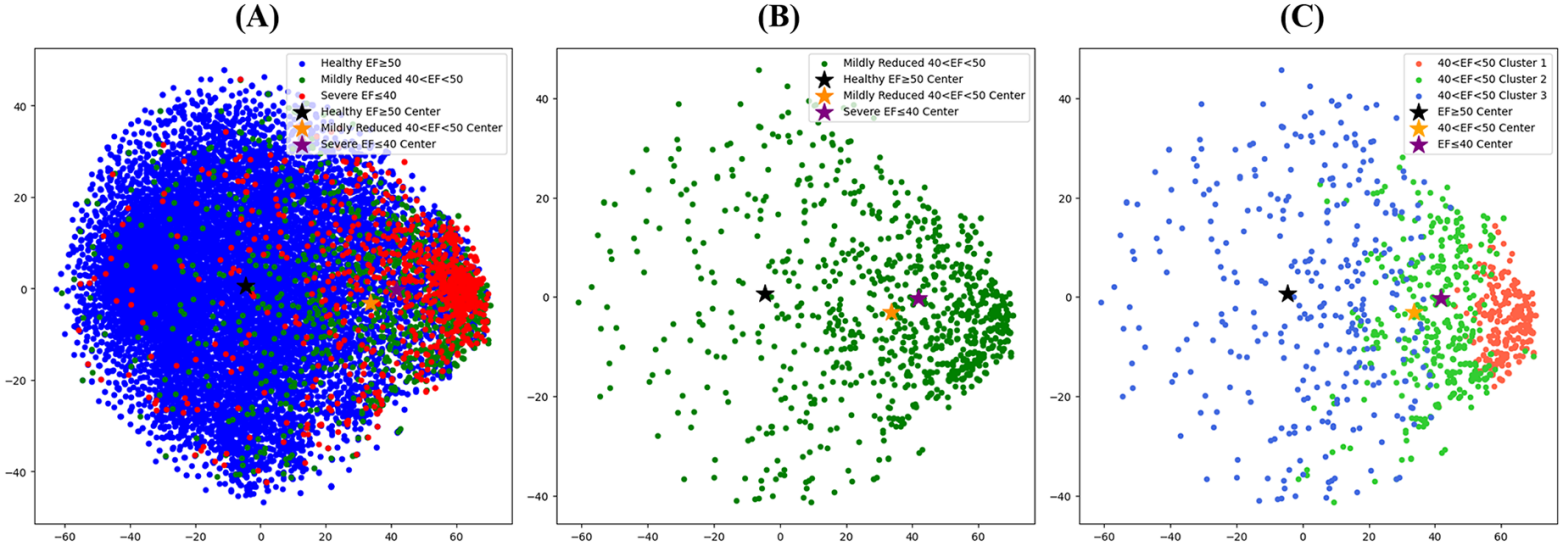
T-SNE visualization of the representation vectors extracted by the AI model. **(A)** T-SNE visualisation of the expression vectors extracted for all holdout test sets for patients with the AI-ECG model trained on the ECG dataset. **(B)** Visualization of representation vectors extracted from only the ECGs corresponding to HFmrEF in the holdout test set. **(C)** Visualization of the HFmrEF expression vector partitioned into three clusters by K-means clustering with a parameter of *k* = 3. AI, artificial intelligence; ECG, electrocardiography; EF, ejection fraction; HFmrEF, heart failure with mildly reduced ejection fraction; HFrEF, heart failure with reduced ejection fraction.

**Figure 4:**
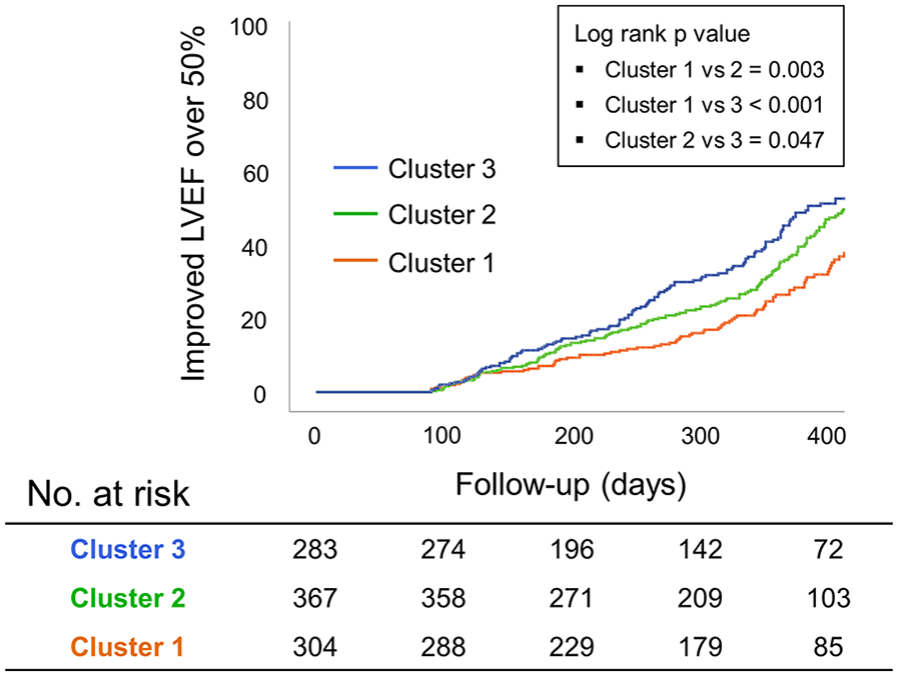
Kaplan–Meier analysis regarding improving LVEFs among the three “Cluster” groups by the AI-estimated Euclidean distance in patients with HFmrEF. AI, artificial intelligence; EF, ejection fraction; LV, left ventricle.

We extracted the representation vectors of the holdout test data using an AI-ECG. Figure 3 shows the transformation of the 256-dimensional representation vectors into two-dimensional data by applying T-distributed Stochastic Neighbor Embedding (T-SNE). The black, yellow, and purple stars in Figure 4 represent the mean vectors of patients with normal EF, HFmrEF, and HFrEF, respectively. Among the 13,767 holdout test dataset shown in Figure 3A, patients with normal EF occupied the largest area with 11,910 instances. With 984 instances, HFmrEF was more widely distributed because it shares characteristics with patients with normal EF and those with HFrEF. However, the HFmrEF is slightly skewed toward the HFrEF region. This indicates a higher similarity between the HFmrEF and HFrEF ECGs. The mean vector of the HFrEF of 873 patients was positioned furthest from the mean vector of the normal EF of patients and was predominantly distributed to the right. Figure 3B shows a visualisation of the representation vector extracted only from the ECGs corresponding to HFmrEF in the holdout test set. Figure 3C shows the partitioning of the HFmrEF representation vector into three clusters using k-means clustering with a parameter of *k* = 3. Notably, Cluster 1 resides in close proximity to the centre of the HFrEF vector, indicating the composition of patients with compromised health status. Conversely, Cluster 3, positioned closest to the centre of the normal EF vector, encompassed patients exhibiting relatively favourable health conditions. Cluster 2 was positioned between Clusters 1 and 3 but exhibited a skewed tendency toward Cluster 1. We applied the *k*-means clustering we learned here to follow up the external validation data and obtained intriguing results. An analysis of these findings is presented in Figure 4.

### Characteristics of patients with mildly reduced HF according to the AI-estimated Euclidean distance from the ECGs of patients with HFrEF and preserved EF

We analysed ECG features, such as PR, QT, QTc interval, QRS duration, and P, R, and T axes using the AI-estimated Euclidean distance to identify specific correlations with the “Severity by Euclidean distance” of the patients with HFmrEF. As a result, the QRS duration (*p* = 0.001), QT interval (*p* = 0.045), and QTc interval (*p* = 0.041) increased significantly with increasing “Severity by Euclidean distance” (Supplementary Figure 3).

We performed additional analysis of the three cluster groups to determine whether AI-ECG could predict the LVEF in patients with HFmrEF. In this cohort, the medication history of drugs proven to improve the prognosis in patients with HF was investigated, and the medications taken continuously for at least 3 months are presented in Supplementary Table 1. As a result, renin-angiotensin-aldosterone inhibitors were taken the most in Cluster 1. However, there was no significant difference in the proportion of beta-blockers, mineralo-receptor antagonists, and sodium-glucose cotransporter 2 inhibitors in each cluster. This meant that the medications did not show a consistently higher rate in either cluster. Kaplan–Meier analysis where the LVEF was improved by ≥50% on follow-up echocardiography revealed that Cluster 1 had poor results compared with Cluster 2 (*p* = 0.003) or Cluster 3 (*p* < 0.001) (Figure 4).

## Discussion

This study developed and validated AI-ECG to identify the types of HF in patients using 12-lead ECG; this demonstrated a reasonable performance in identifying patients with HFrEF and those with normal EF. Because the performance in identifying patients with HFmrEF was relatively low compared with that in identifying patients with other types of HF, we sought ECG characteristics using the AI-estimated Euclidean distance in these patients. It was revealed that the QRS duration, QT interval, and QTc interval showed significant changes according to severity. Based on these results, AI-ECG may be used to predict the prognosis of patients with HFmrEF.

### Clinical applications of developing a model that identifies HFmrEF

Since the European Society of Cardiology Heart Failure Guidelines first recognised HFmrEF as an independent entity, different from HFrEF and HFpEF, in 2016, HFmrEF has been regarded as a major disease entity in all types of HF (16). It also poses a significant burden on global healthcare as its prevalence increases (17). As mentioned above, patients with HFmrEF have heterogeneous characteristics, and many previous studies on HFmrEF have not identified consistent similarities between the characteristics of HFmrEF and those of either HFrEF or HFpEF (18). Furthermore, patients with HFmrEF tend to exhibit a change in their phenotypes, which is represented by LVEFs over time, more predominantly than those with other types of HF (19). Therefore, developing a model that detects the degree of cardiac dysfunction using AI-ECG could help manage these patients (20).

Until now, several studies have been conducted to detect cardiac contractile dysfunction using AI (9, 10, 21, 22), Attia et al. (9) revealed an AI-based model for identifying patients with LV systolic dysfunction (defined as LVEF under 35%) using ECG and echocardiography. Its performance had an AUC of 0.93. After 3.4 years of follow-up, patients with baseline positive AI-ECG and negative echocardiography had significantly higher rates of LV systolic dysfunction. Another study by Zhang et al. (10) reported the AI model to detect patients with congestive HF, and the results showed good performance and accuracy, sensitivity, and specificity reached 94.97%, 89.38%, and 99.50%, respectively. A study by Yao et al. (21) designed an AI model for identifying patients with lower LVEF under 50% by randomized clinical trial. This model showed that the intervention group (the patients who used this AI model) had a higher rate of low EF (2.1% vs. 1.6% in control, odds ratio 1.32 (1.01–1.61) compared with the control group. Similarly, Vaid et al. (22) showed AI-ECG to predict cardiac dysfunction from ECG, and it demonstrated strong performance in detecting both right ventricular and LV systolic dysfunction (LVEF ≤40%), with AUCs of 0.84 and 0.94, respectively, across internal and external databases. All of those studies are consistent with our study in that they used DNN with numerous ECG data in a real-world population, predicted ventricular dysfunction defined by echocardiography, and showed good performance of AI model. However, no clinical trials have been conducted to detect HFmrEF. HFmrEF, unlike other HF phenotypes, has a narrow range with a LVEF of 41%–49%, and has complex and heterogeneous patient characteristics. Patients with HFmrEF have fewer complaints of dyspnoea or chest discomfort than those with HFrEF at the time of diagnosis. For this reason, predicting these patients and their prognosis could be a clinical burden. Therefore, it might be important to screen asymptomatic patients for HFmrEF and to prepare them for the possible manifestations of cardiac dysfunction. This AI-based trial could predict the prognosis of patients with HFmrEF, leading to appropriate medical strategies. Furthermore, its application in clinical practice would be utilized by longitudinal monitoring of patients, which would involve quick and precise detection of cardiac problems (23–26).

Given the increasing burden of HF on healthcare system, it is worth valuable to discuss the economic benefits of adoping AI-ECG in patients with HFmrEF. Most of the economic costs of HF patients are attributable to inpatient hospitalization costs (27). About 800,000 heart failure patients experienced hospitalization per year in the United States, and the cost per person was about $19,000. Considering the prevalence of HFmrEF (10%–25%), it is estimated that about 80,000–200,000 hospitalized patients were present. If the clinical effect of AI-ECG can reduce the hospitalization rate of HFmrEF patients by 10%, the expected economic savings per year can be estimated at about $19 million to $28 million.

### Performance of AI-ECG

AI-ECG in this study revealed an overall accuracy of 87.3% (95% CI: 86.4–89.3) and AUCs of >0.80 for each phenotype of HF. Based on these results, this model demonstrated a favourable performance. Other clinical trials predicting HF subtypes have shown comparable results. A previous study predicted LV systolic dysfunction as an LVEF of <35% in the normal population with an AUC of 0.93 (9). Another study achieved an AUC of 0.87 with a deep-learning model to detect HFpEF in the normal population (8). In our study, different detectability results were obtained for each type of HF. The AUCs for detecting an EF of ≤40% and ≥50% were 0.875 and 0.870, respectively. However, to detect an EF between 41% and 49%, the number of patients with HFmrEF was relatively low (0.824). We could not directly compare the performance of our model in detecting HFmrEF with that of the others, as there have been no other reference studies using AI involving patients with HFmrEF. However, it was postulated that since patients with HFmrEF have more heterogeneous, complex, and diverse aetiologies, it could be more difficult to detect patients with HFmrEF than those with other types of HF using AI-ECG (28). The results of the scarce pattern of T-SNE visualisation in patients with HFmrEF also reflect these features. Although detectability in the HFmrEF group was not effective, this model may provide important clues regarding the patient's clinical prognosis. For example, even in patients with HFmrEF with the same range of EF, AI-ECG could detect “those with the possibility of upcoming ‘HFrEF’ for one group and ‘those with normal EF’ for another group”. If patients are predicted to be in the HFrEF group and have more electrocardiographic characteristics of HFrEF than those not predicted, their clinical characteristics may be similar to those of patients with HFrEF. However, further studies with more detailed designs are required to verify this hypothesis.

### ECG characteristics according to the AI-estimated Euclidean distance

In our study, the ability of AI-ECG to distinguish the ECG pattern in each HF phenotype was generally acceptable, except for HFmrEF. As a result of analysing the characteristics according to the AI-predicted EF Euclidean distance of patients with HFrEF and those with a normal range of EF, abnormal ECG findings such as a prolonged QRS duration, QT interval, and QTc interval, were closely correlated with those in patients predicted to have a condition closer to HFrEF. Conversely, a shorter QRS duration, QT interval, and QTc interval were related to a predicted ECG finding closer to that of the normal population. Previous studies correlate several ECG features with different types of HF. Purnasidha et al. created a scoring system to predict HFrEF based on ECG characteristics. A longer QRS duration (>100 ms) and QT interval are associated with HF with systolic dysfunction (29), and these findings support our observations, along with left atrial hypertrophy and right bundle branch block. Other studies also showed an association of a QRS duration of >100 ms with a reduced EF of <45% (30) and prolonged QTc interval with adverse outcomes, such as long-term mortality (31). Based on the ECG characteristics, our findings in the HFmrEF group (scattered pattern of T-SNE and lower performance in detecting the group) reflect the non-unified characteristics of patients with HFmrEF. Applying the AI-based model in patients with HFmrEF could have great clinical applicability as it can predict the prognosis of this patient cohort and the responses, such as drug response and cardiovascular outcomes.

### Limitations

This study had some limitations. First, this was a retrospective study, and there is a possibility of selection bias. To minimise this limitation, we collected data from approximately 100,000 ECGs from over 50,000 patients. Second, the study population was recruited from a single centre in South Korea. Further studies involving more institutions are required to validate this model. Third, given the inherent limitations of deep neural networks, they have structural limitations in creating causal relationships. To minimise these limitations, training, validation, and holdout testing were performed using large amounts of refined raw digital ECG data. Fourth, an echocardiographic assessment of the entire population was not performed using the same equipment, and not all analyses of LVEF were performed using Simpson's method, which might have produced inconsistencies in echocardiographic estimations. Fifth, in the analysis to verify the predictability of LVEF in clustering patients who underwent follow-up ECG, a consistent follow-up duration of ECG was not shown for each patient because of its retrospective nature. Future prospective studies should address these limitations. Additionally, to enhance the study's robustness, the training, validation, and holdout test sets were segmented based on different time periods. As a result, while the proportions of normal EF in the datasets are similar, the training set contains a larger number of normal EF, leading to clinical bias, as observed in Table 1.

## Conclusions

AI-ECG could be used to identify patients with HFmrEF and predict future cardiac contractility based on ECG characteristics. Further prospective studies are required to examine the feasibility of its use in clinical practice.

## Data Availability

The datasets presented in this article are not readily available due to privacy concerns. Requests to access the datasets should be directed to the corresponding author.
