# Supplementary figures and images for: Electrocardiography-based artificial intelligence predicts the upcoming future of heart failure with mildly reduced ejection fraction
Source: Front Cardiovasc Med. 2025 Feb 10;12:1418914. doi: 10.3389/fcvm.2025.1418914 (PMC11847893; doi:10.3389/fcvm.2025.1418914)

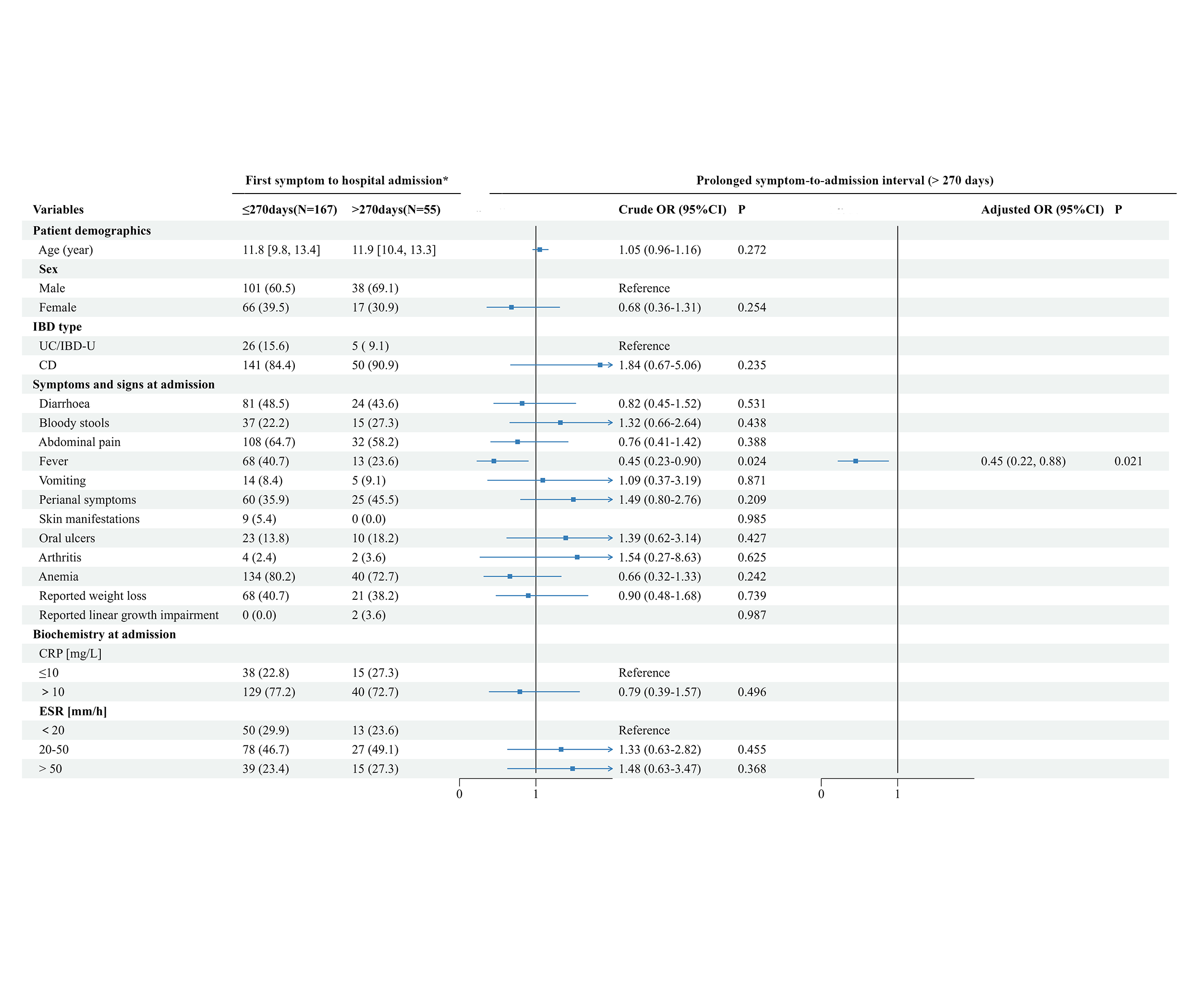

Supplement: Supplementary file 1 [file Image1.tif]

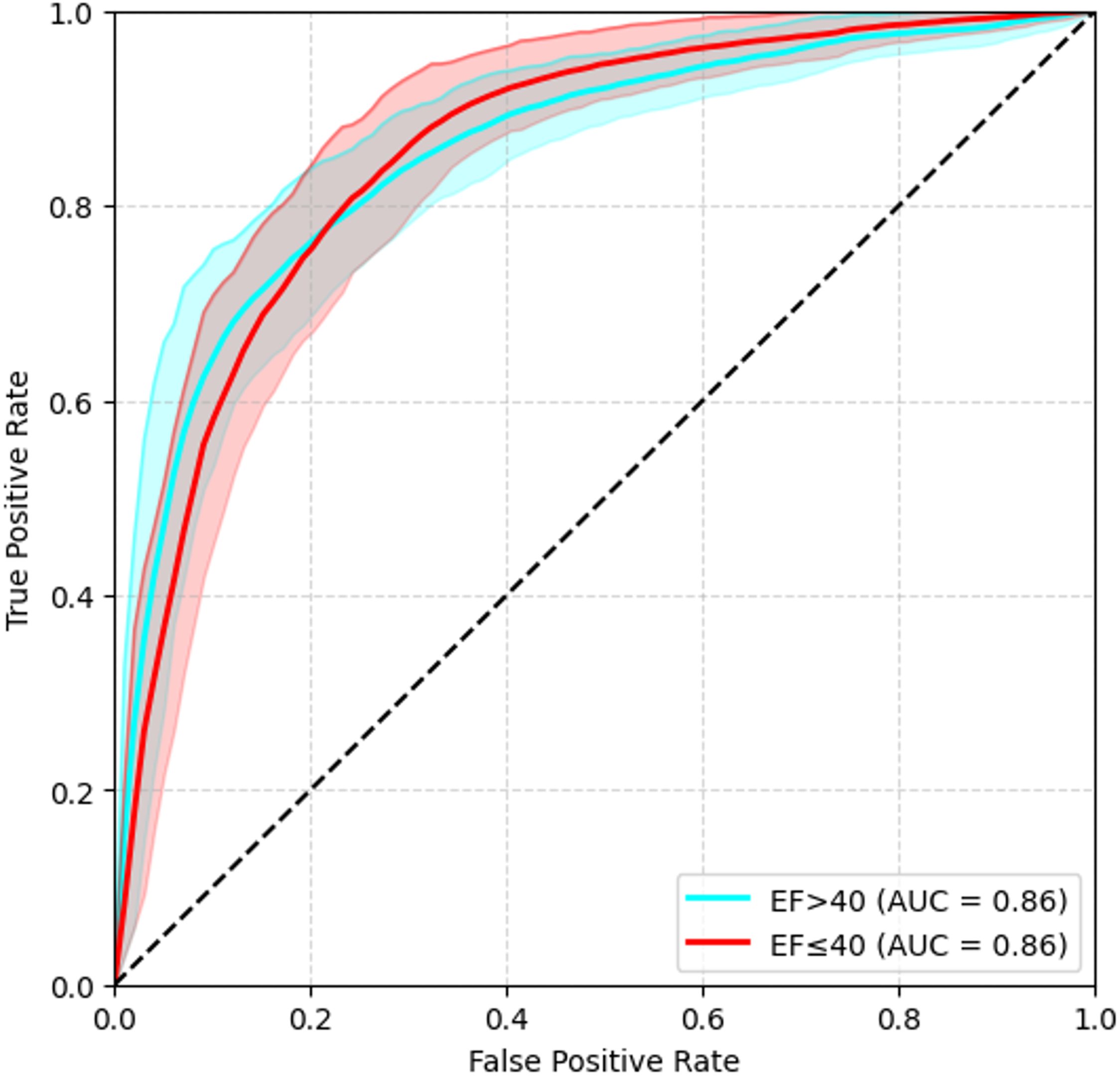

Supplement: Supplementary file 2 [file Image2.tif]

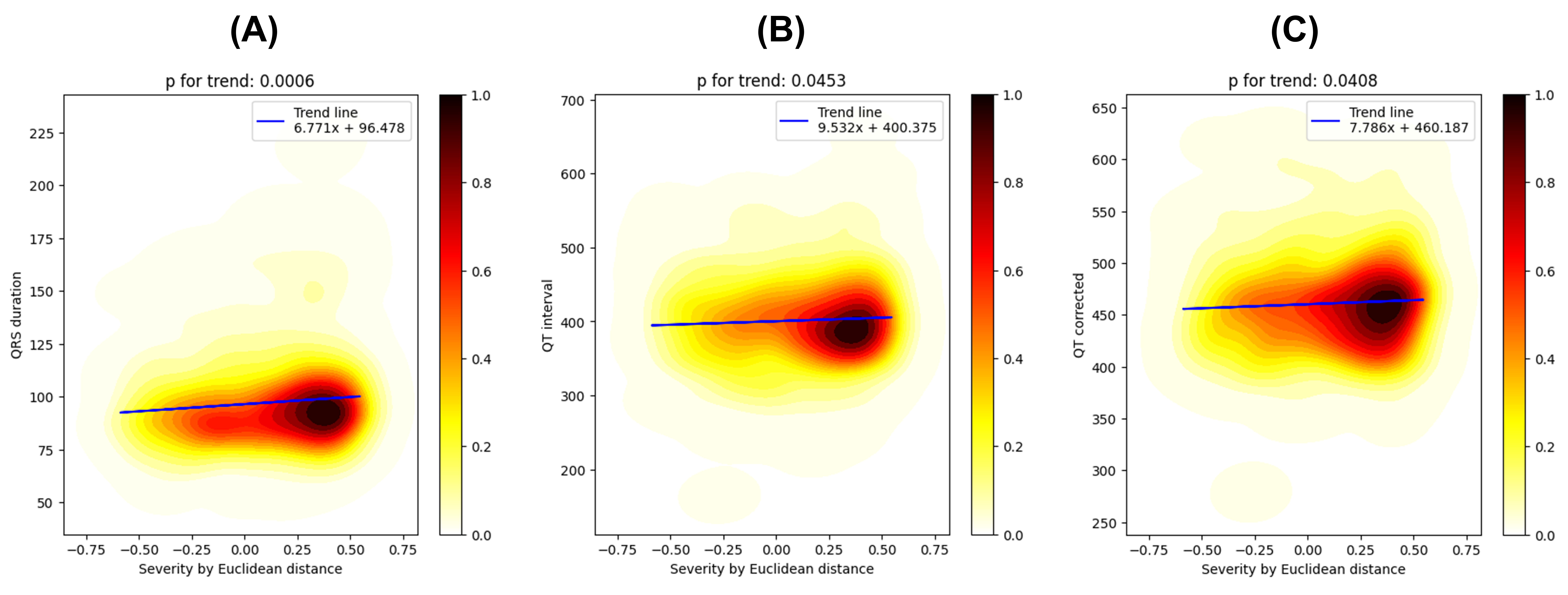

Supplement: Supplementary file 3 [file Image3.tif]
