# Supplementary material for: Electrocardiography-based artificial intelligence predicts the upcoming future of heart failure with mildly reduced ejection fraction
Source: Front Cardiovasc Med. 2025 Feb 10;12:1418914. doi: 10.3389/fcvm.2025.1418914 (PMC11847893; doi:10.3389/fcvm.2025.1418914)
Supplement: Supplementary file 4 [file Table1.docx]

Supplementary Material

***Data preprocessing***

**Supplemenatary Figure 1** illustrates the proposed model. We introduced a simple automatic labelling method to determine the location of R peaks. First, we found values greater than the median of the mean and maximum values of lead-1. Subsequently, the number of consecutive sets of values was counted; this is the number of R peaks that can be used to calculate the approximate heart rate. We found that some data were labelled with heart rates that were difficult to obtain. To select reliable labelled data, we extracted data corresponding to a heart rate of 40-135 beats per minute, which is generally possible based on the resting heart rate of 60-100 beats per minute for a typical adult. In this study, we use 10-s data, including data with 7-22 peaks. We trained the LightGBM (1) using the extracted data and label set. After the input data were provided, we first predicted the number of R peaks using the LightGBM model. We then divided the data evenly into numbers of R peaks. Next, the maximum value for each of the divided ranges was determined. Finally, we cut the data until we found two R peaks on each side of the middle R peak and interpolated the data for use as the input of the transformer model. (2).

***Transformers for severity by Euclidean distance in*** ***heart failure with mildly reduced ejection fraction (HFmrEF)***

The Transformer model (2) was originally proposed to capture the sequential characteristics of natural languages. BERT (3) uses transformer encoders to extract natural language representations, and GPT (4) uses transformer decoders to innovate in natural language processing. Natural language and time-series data share the common characteristics of sequential relationships. As a result, the transformer model also exhibits strong performance in processing time series data. (5),(6),(7) In this study, we extracted the representation between electrocardiography (ECG) and ejection fraction (EF) based on transformer encoders and calculated the similarity score of HFmrEF, considering normal EF and heart failure with reduced ejection fraction (HFrEF).

The preprocessed data pass through a 1D convolution layer to learn local contextual information and are augmented with positional encoding to inject sequence information. The processed data then undergo layer normalisation (8) and are projected into query $Q\in\mathbb{R}^{s\times d}$, key $K\in\mathbb{R}^{s\times d}$, and value $V\in\mathbb{R}^{s\times d}$ to be used as inputs for the Transformer encoder. In this study, we defined s as the length of the preprocessed ECG signals, which was 1,600, and d as the size of the hidden dimension, which was set to 512. The Transformer encoder consists of multi-head attention, layer normalisation, and convolutional feedforward layers. The multi-head attention in the transformer encoder is defined by the following equation:

$$MH\left( Q,K,V \right)=Concat({\mathbb{A}\left( Q,K,V \right)}_{1},\ldots{\mathbb{A}\left( Q,K,V \right)}_{h})W$$

$$\mathbb{A}\left( Q,K,V \right)=softmax(\frac{QK^{T}}{\sqrt{d}})V$$

The softmax function of the matrix multiplication of queries and keys captures important parts only from the input ECG and amplifies them by multiplying them by the values. The convolutional feed-forward layer, consisting of two 1D convolutions, is designed to learn relationships with neighbouring sequences and is defined as:

$$CFF\left( x \right)=\left( \sum_{j=0}^{d} W_{2}\odot\left( \sum_{i=0}^{4d} W_{1}\odot X^{T}+b_{1} \right)+b_{2} \right)^{T}$$

where ‘⊙’ is the cross-correlation operator,(9) and the transformer encoder structure is repeated four times. The projection and representation vector extraction layers, based on the linear layer, project the hidden dimension and output 256 representation vectors from 1,600 sequences. The representation vectors were trained using the triplet loss,(10) defined by the following equation:

$$L\left( A, P, N \right)=Max(0, \left\| A-P \right\|_{2}-\left\| A-N \right\|_{2}+\alpha)$$

where A is the Anchor and is the data currently being trained. P is a Positive Sample, another sample belonging to the same class as the Anchor, and N is a Negative Sample, a sample belonging to a class different from the Anchor. We determined the positive and negative samples using three classes: HFmrEF, normal EF, and HFrEF. Using the triplet loss, the Euclidean distance between the Anchor and Positive Samples decreases, and the Euclidean distance between the Anchor and Negative Samples increases. The anchor representation vector trained with triplet loss was passed through three linear layers to classify HFmrEF, normal EF, and HFrEF. This training approach quantifies the severity using the Euclidean distance between the extracted representation vectors.

**Supplementary Table**

**Supplementary Table 1.** Medications for each ‘Cluster’ group

|  | All  (n=1,134) | Cluster 1  (n=445) | Cluster 2  (n=358) | Cluster 3  (n=331) | P-value |
| --- | --- | --- | --- | --- | --- |
| RAASi, n (%) | 639 (67.0) | 230 (75.7) | 243 (66.2) | 166 (58.7) | <0.001 |
| Beta blocker, n (%) | 710 (74.4) | 240 (78.9) | 264 (71.9) | 206 (72.8) | 0.088 |
| MRA, n (%) | 309 (32.4) | 96 (31.6) | 109 (29.7) | 104 (36.7) | 0.153 |
| SGLT2i, n (%) | 95 (10.0) | 32 (10.5) | 36 (9.8) | 27 (9.5) | 0.917 |

RAASi, renin-angiotensin-aldosterone system inhibitor; MRA, mineralo receptor antagonist; SGLT2i, sodium glucose cotransporter 2 inhibitor.

**Supplementary Figure**

**Supplementary Figure 1.** Description of AI-ECG for left ventricular ejection fraction in patients with heart failure. AI, artificial intelligence; ECG, electrocardiography.

**Supplementary Figure 2.** AI model performance and ROC curve in all patients except HFmrEF. AI, artificial intelligence; ROC, receiver operating characteristic; HFmrEF, heart failure with mildly reduced ejection fraction.

**Supplementary Figure 3.** Characteristics of patients with HFmrEF according to AI that were used to estimate the Euclidean distance from HFrEF to normal EF. AI, artificial intelligence; HFmrEF, heart failure with mildly reduced ejection fraction; HFrEF, heart failure with reduced ejection fraction.

**References**

1. Ke G, Meng Q, Finley T, Wang T, Chen W, Ma W, Ye Q, Liu T-Y. Lightgbm: A highly efficient gradient boosting decision tree. Advances in neural information processing systems. (2017) 30:3149–3157.

2. Ashish Vaswani, Noam Shazeer, Niki Parmar, Jakob Uszkoreit, Llion Jones, Aidan N. Gomez, et al. Attention is all you need. Advances in neural information processing systems. (2017) 30:6000–6010. doi: [10.48550/arXiv.1706.03762](https://doi.org/10.48550/arXiv.1706.03762)

3. Devlin J, Chang M-W, Lee K, Toutanova K. Bert: Pre-training of deep bidirectional transformers for language understanding. arXiv preprint arXiv:1810.04805. (2018). doi: [10.48550/arXiv.1810.04805](https://doi.org/10.48550/arXiv.1810.04805)

4. Brown T, Mann B, Ryder N, Subbiah M, Kaplan JD, Dhariwal P, Neelakantan A, Shyam P, Sastry G, Askell A. Language models are few-shot learners. Advances in neural information processing systems. (2020) 33:1877-1901. doi: [10.48550/arXiv.2005.14165](https://doi.org/10.48550/arXiv.2005.14165)

5. Wu H, Xu J, Wang J, Long M. Autoformer: Decomposition transformers with auto-correlation for long-term series forecasting. Advances in Neural Information Processing Systems. (2021) 34:22419-22430. doi: [10.48550/arXiv.2106.13008](https://doi.org/10.48550/arXiv.2106.13008)

6. Zhou T, Ma Z, Wen Q, Wang X, Sun L, Jin R. Fedformer: Frequency enhanced decomposed transformer for long-term series forecasting. Paper/Poster presented at: International Conference on Machine Learning. (2022).

7. Lee, S., Hong, J., Liu, L., & Choi, W. TS-Fastformer: Fast Transformer for Time-Series Forecasting. ACM Transactions on Intelligent Systems and Technology. (2024) 15(2):1-20. doi: [10.1145/3630637](https://doi.org/10.1145/3630637)

8. Ba JL, Kiros JR, Hinton GE. Layer normalization. arXiv preprint arXiv:1607.06450. (2016). Doi: [10.48550/arXiv.1607.06450](https://doi.org/10.48550/arXiv.1607.06450)

9. Rhudy M, Bucci B, Vipperman J, Allanach J, Abraham B. Microphone array analysis methods using cross-correlations. In: ASME International Mechanical Engineering Congress and Exposition. 2009. P. 281-8. Abstract 43888.

10. Schroff F, Kalenichenko D, Philbin J. Facenet: A unified embedding for face recognition and clustering. In: Proceedings of the IEEE conference on computer vision and pattern recognition (2015). p.815-823.
